# Stroke risk in younger age groups is increasing and requires targeted prevention

**DOI:** 10.1371/journal.pmed.1005232

**Published:** 2026-08-26

**Authors:** Iain J. Marshall, Charles D. A. Wolfe, Matthew D. L. O‘Connell

**Affiliations:** Department of Population Health Sciences, King’s College London, London, United Kingdom

## Abstract

In this Perspective, Iain Marshall and colleagues discuss a new study that suggests stroke risk may be increasing among younger age groups, highlighting new emerging risks and discussing the need for more accurate risk algorithms and novel targeted prevention programmes.

## Teaser

While global stroke incidence has reduced considerably in recent years, a new study suggests that stroke risk may be increasing among younger age groups. Worryingly, the main drivers are well-established risk factors with effective treatments, but new risks are emerging. Development of more accurate risk algorithms and novel targeted prevention programmes are needed to reduce this risk.

Stroke remains a leading cause of death and adult disability worldwide and was the third leading cause of death (~7 million deaths) and the fourth leading cause of disability (~160 million Disability Adjusted Life Years) globally in 2021 [1]. Stroke’s global health impact requires policymakers, clinicians and stroke survivors to reset the agenda for prevention in novel ways to reduce poor outcomes after stroke. The Global Burden of Disease (GBD) studies have shown an encouraging 40% decline in stroke risk over the last 40 years, although this is largely driven by a decline in stroke incidence among older adults [1]. In the UK, local population-based studies have shown a tailing off in this decline and small but significant rises in younger age groups, particularly ethnic minority groups [2]. This, along with a stark projected 30% increasing risk over the next few decades—mainly because of ageing populations—paints an alarming backdrop for a disease we have for too long considered preventable through the management of traditional risk factors [3]. In addition, ageing populations and increased cardiovascular disease risk are being observed in low- and middle-income countries (LMICs), further emphasising the need for research to better understand changing trends in stroke risk in different settings that can inform targeted interventions [1,2].

Few countries have the advantage of utilising multiple national datasets to estimate risk over time. However, in a recent study published in *PLOS Medicine*, Li and colleagues [4] utilise a novel methodology by employing real-world, linked data sources in the UK to estimate risk over a relatively short time frame before and after the COVID-19 pandemic. While this time window is relatively small, the findings are highly relevant for policymakers, health service delivery, and populations at highest risk. The use of linked primary care and hospital data is particularly important when a significant proportion of people with stroke are not hospitalised, for example during the pandemic. Compared with a single source, linkage with multiple sources also helps to minimise bias related to changes in patient behaviour and reduces the chances of missing people with stroke. Together, the estimates derived from routinely collected information from primary care, death certificates, and national stroke audit data show a doubling of stroke risk in the under 55-year-old age group over the last 5 years, partly because of rising incidence rates in ethnic minority populations [4]. However, within these trends, the effect of the pandemic is difficult to disentangle from other causes of increased stroke risk and in the study by Li and colleagues the all-age increase in risk was only 10%, probably explained by reduced admissions to hospital during this period [4,5]. Similarly, the GBD and other studies showed a stabilisation of stroke incidence rates during the pandemic years illustrating the likelihood that data sources were mainly registering those admitted to hospital [6].

It is well established that ‘traditional’ cardiovascular disease risk factors substantially increase stroke risk, are common, and have evidence-based treatments. There has been a particular focus on hypertension, diabetes, and rising obesity around the world, and national surveys show declines in the prevalence of smoking, hypertension, raised cholesterol and body mass index while atrial fibrillation rates (which influence stroke risk) have increased [7]. The decline in stroke risk to date has largely been attributed to better control of such risk factors, although this has never been fully substantiated as other emerging and non-clinical risk factors have not been considered when interpreting trends in risk.

Emerging risks (e.g., air pollution, climate change-related extreme heat, and early-life disadvantage) have been less considered and will come to the forefront, as will the widening of inequalities in risk particularly in deprived populations—where poverty and low socioeconomic status are notable risk factors—and in ethnic minority communities [8,9]. Each country or region will have different risk factor profiles and trends, such that prevention policies and services should ideally be tailored at a local level to potentially maximise prevention programmes.

The GBD studies estimate that decreases in behavioural, environmental, and occupational risks have largely offset the effects of population growth and ageing, in relation to trends in absolute burden. Conversely, the combination of increasing metabolic risks and population ageing will probably continue to drive the increasing trends in non-communicable diseases at the global level, which presents both a public health challenge and opportunity.

The current paradigm for stroke is that it is a disease linked with ageing and that traditional risk factor control is central to reducing the burden of stroke. However, the divergent findings in younger people and in ethnic minorities suggest we need a rethink for risk assessment and primary prevention. Understanding how to engage with younger adults in their 20s, 30s, and 40s is paramount; for example, designing methods to improve risk factor detection and adherence to preventive treatments across cultures, ethnicities and communities. Research gaps include understanding much more about behaviours in different groups of the population, how to improve the uptake of medications and ongoing monitoring, and ensuring that diverse communities are engaged with when prevention programmes are implemented.

An obvious area for adjustment is the cardiovascular risk algorithms used to decide upon preventive treatment, which (nearly) universally score under 40-year-olds as low risk, since they are so heavily age weighted [10]. Further exploration of increasing the number of clinical and socioeconomic variables in the algorithms for people in their 20s and 30s is therefore required. Expanding detection and treatment of cardiovascular risk factors in this way will require a huge expansion of primary prevention programmes [11,12].

As Li and colleagues suggest [4], tailored interventions might also be needed to narrow the long-standing inequalities by ethnicity, requiring targeted public health education to enhance health literacy, as well as culturally sensitive screening strategies to improve uptake of risk factor screening and adherence to treatment [13].

Despite the presence of effective medications, stroke risk has approximately doubled among adults younger than 55 years in the UK over the past decade [4]. This is not encouraging, and points to the need for a clearer way forward for early diagnosis and adherence to medication, as well as strategies to tackle known and newly emerging risks such as poverty, climate change, and worsening air quality.

## Abbreviations

GBD: Global Burden of Disease
LMICs: low- and middle-income countries
